# Dark tetrad personality traits also play a role in bullying victimization

**DOI:** 10.3389/fpsyg.2022.984744

**Published:** 2022-10-14

**Authors:** David Pineda, Pilar Rico-Bordera, Ana Martínez-Martínez, Manuel Galán, José A. Piqueras

**Affiliations:** ^1^Forensic Psychology Unit, Department of Health Psychology, Centre of Applied Psychology, Miguel Hernández University of Elche, Elche, Spain; ^2^Department of Psychology, Faculty of Health Sciences, Catholic University of Murcia, Murcia, Spain

**Keywords:** bullying, victimization, Dark Tetrad, narcissism, machiavellianism, psychopathy, sadism

## Abstract

Bullying refers to physical and/or psychological mistreatment or abuse by one individual or group toward another individual or group. Bullying is widespread in our society and carries considerable negative consequences. This phenomenon is caused by multiple factors, which include personality. Much more attention has been paid to the study of the perpetrators' negative personality traits than the victims. Several studies have examined the relationship between these traits—the Dark Triad or Dark Tetrad—and being a victim of bullying (or mobbing) in adults, especially in the workplace. However, only two studies have been located that have studied these relationships in adolescents. Therefore, this study aimed to analyze the relationship between being a victim of bullying and the ark Tetrad traits, delving into the specific contribution of Machiavellianism, narcissism, psychopathy, and sadism in victims of bullying in Spanish adolescents. A cross-sectional study was carried out by administering the Short Dark Triad, the Assessment of Sadistic Personality, and the Peer Bullying Questionnaire to 393 adolescents aged 12–18 years (*M* = 14.18; *SD* = 2.52; 53.7% male). The Dark Tetrad traits predicted the victimization variables in the seven models analyzed, with the verbal abuse model being the model with the largest contribution. Of the four dark traits, sadism stands out as the trait with the highest specific contribution. Our results indicate, despite not implying a causal relationship, that those people with high scores in the Dark Tetrad traits tend to be more victimized by bullying. Knowing the personality traits of the bullying perpetrators and their victims, practitioners will have a complete picture of the personality variables that play a role in preventing bullying and its associated victimization.

## Introduction

### Bullying

Bullying, or peer bullying, is a type of aggression that refers to physical and/or psychological mistreatment or abuse by one individual or group toward another individual. This type of aggression can occur in different contexts (i.e., inside or outside the school, face-to-face, or online) and in many different ways (e.g., directly aggressing, threatening, or verbally abusing), making it a widespread phenomenon (Olweus, 1978; Magaz et al., 2016; Sorrentino et al., 2019). Therefore, with this variety of possibilities, the bullying prevalence rates are variable between studies depending on the measures used and the sample considered. However, the research on this topic states that up to 40% of children and youth have been involved in bullying behaviors as victims (e.g., Zych et al., 2016; Sorrentino et al., 2019; Biswas et al., 2020; Fuentes Chacón et al., 2020; Larrain and Garaigordobil, 2020).

Considering these high prevalence rates of bullying victimization, we must consider the consequences of being victimized by a bully. Suffering from bullying negatively affects the physical, mental, and socio-emotional health, as well as the wellbeing of the children who are bullied (Bond et al., 2001; Camerini et al., 2020). These consequences can range from depression or anxiety symptoms to behavioral disorders, substance abuse, or even suicidal behaviors (e.g., Reijntjes et al., 2010; Holt et al., 2015; De Lara, 2018).

Recent research has focused on analyzing the risk factors most associated with both experiencing and perpetrating bullying, which aids in the design of more targeted intervention programs (e.g., Huang et al., 2019; Ng et al., 2020; Martínez-Martínez et al., 2021). On the one hand, some of the risk factors that make someone more prone to suffering bullying are being male, having a disability or other health problems, having a low mood and poor self-perception, feeling lonely and having feelings of dissatisfaction with life, having few cognitive skills, having a poor relationship with peers or parents, having few economic resources and perceiving a lower quality of life, having a sexual orientation other than heterosexual, etc. (Puértolas Jiménez and Montiel Juan, 2017; Fuentes Chacón et al., 2020; Kahle, 2020).

On the other hand, the main characteristics that have been associated with the perpetration of bullying behaviors are high levels of anger and self-esteem, perceived high empathy, exposure to pornography, traditional masculinity, low levels of social and parental support, higher levels of school attachment, alcohol consumption, lack of emotional control, etc. (Leemis et al., 2019; Qian et al., 2020). Furthermore, individual personality differences have also been shown to play an important role in involvement in bullying situations, both as a bully and as a victim (e.g., Mitsopoulou and Giovazolias, 2015; Zhang et al., 2021).

### Bullying and personality (the Dark Tetrad traits)

Personality traits like honesty-humility, emotionality, agreeableness, and openness to experience were negatively related to bullying perpetration (Pronk et al., 2021). However, different results have appeared while studying different populations. For example, Volk et al. (2018) pointed out that those participants with lower scores in honesty-humility and conscientiousness were more prone to perpetrating bullying, while those with lower scores in extraversion suffered from it. Notwithstanding, these authors also found a more complex relationship between personality and bullying in their Chinese sample, highlighting the importance of studying these connections in different cultures.

However, when personality is used to explain antisocial or criminal behaviors, a group of traits tends to predict these behaviors beyond general personality models. This is the Dark Tetrad of personality. First described as The Dark Triad by Paulhus and Williams (2002), three “dark” personality traits were described: subclinical psychopathy refers to a pattern of callousness and impulsivity; machiavellianism, which refers to the manipulation and lack of morality; and subclinical narcissism, which would broadly refer to a grandiose sense of identity with the necessity of admiration (Jones and Paulhus, 2014). With the increase of the investigation into these traits, the trait of everyday sadism was included. This trait would briefly describe a person who derives pleasure or joy from others' suffering (Chabrol et al., 2009; O'Meara et al., 2011).

On the one hand, studies that have analyzed the relationships between the negative personality traits of bullies have found positive connections. This indicates that those with high scores in the dark personality traits tend to be more involved in performance bullying behaviors (Goodboy and Martin, 2015). Specifically, the Dark Triad trait that predicted bullying the strongest was mainly psychopathy (Goodboy and Martin, 2015; Gul and Fatima, 2016). In contrast, while looking at cyberbullying behaviors, two of the three Dark Triad traits tend to predict them, with those with higher scores in machiavellianism and psychopathy being more involved in these behaviors (Aguilar Cumbicus and Resett, 2002). When looking at the four traits of the Dark Tetrad in conjunction with other sociodemographic variables and the Big Five traits, psychopathy, machiavellianism, and sadism appeared to predict bullying (Van Geel et al., 2017).

On the other hand, the negative personality traits have not only shown a predictive ability for the perpetration of antisocial and criminal behavior but from being bullied and victimized by it (e.g., Hayes et al., 2021; Pineda et al., 2021a; Pineda et al.^1^). In the bullying context, most studies have been developed in work contexts to study mobbing victimization (i.e., bullying victimization in the workplace). Previous studies have found mixed results regarding the most decisive trait predicting workplace bullying victimization, but all of them agree that machiavellianism does predict this victimization (Linton and Power, 2013; Parker, 2019; Fernández-del-Río et al., 2021).

To our knowledge, only two studies have examined these relationships between dark personality traits and bullying victimization in adolescents. One of them (Gul and Fatima, 2016) was conducted with 479 Pakistani adolescents aged 13–18 years (*M* = 15.11; *SD* = 1.24; 245 male), and an instrument with three scales was used to assess victimization: social, physical, and verbal victimization. Gul and Fatima (2016) asserted that only psychopathy from the Dark Triad correlated positively with being a victim of bullying in adolescent girls. However, in this study, regression models showed that none of the three traits predicted bullying victimization in the sample of boys and girls. However, this finding might be explained by the lack of assessment of the direct predictive ability of the Dark Triad traits for victimization since not only are the three Dark Triad traits included in the regression model, but also the effect of bullying perpetration is included in this relationship.

The other study (Boele et al., 2017) was conducted with 1,108 adolescents, mostly Dutch; victimization was measured simply by asking by whom they were bullied. Its results showed no significant correlations with the three Dark Triad traits. As in the previous study, these results could be biased by the type of instrument used.

### The present study

Most studies have focused on analyzing the “dark” personalities of bullies, leaving aside the possible presence of these traits in their victims. Knowing the characteristics of both bullies and victims can facilitate the design of more specific intervention programs, as well as the design of prevention programs to work with personality factors that may predispose to victimization and bullying (Gul and Fatima, 2016; Choi and Park, 2018; Reisen et al., 2019; Martínez-Martínez et al., 2021).

Studies analyzing the relationship between these traits and bullying victimization (in different contexts) suggest that the positive relationship discovered may be because some are also victims or become victims after being bullies (Fanti and Henrich, 2015; Choi and Park, 2018; Reisen et al., 2019). This relationship could also be due to the negative traits of those who perpetrate bullying and do not consider the consequences of being victimized similarly (Foulkes, 2019).

In addition to focusing on victimization, as a particular strength of this study, it is noteworthy that the two studies described above (Gul and Fatima, 2016; Boele et al., 2017) did not measure the trait of everyday sadism, which has shown a crucial predictive ability in victimization situations (Pineda et al., 2020, 2021a). Moreover, none of them assessed the types of bullying victimization separately (i.e., being abused, excluded, threatened, and assaulted face-to-face or online), making it difficult to obtain more specific results.

To fill this gap, the main aim of this study was to analyze the influence of the Dark Tetrad traits on bullying victimization behaviors in a Spanish adolescent sample. Furthermore, we aimed to analyze the specific contributions of each of these four traits (i.e., machiavellianism, narcissism, psychopathy, and sadism) in being victimized by bullying in different ways (i.e., being abused, excluded, threatened, or aggressed) and in different contexts (i.e., face-to-face and online).

Therefore, this is the first study to analyze the predictive ability of the Dark Tetrad traits for adolescent victimization. Following previous literature (albeit with not very consistent results) that assessed these or similar relationships, we expect to obtain a positive correlation between psychopathy (H_1_) and machiavellianism (H_2_), and no significant correlation with narcissism (H_3_). Regarding everyday sadism, although no studies have previously assessed this relationship specifically with adolescents, we hypothesize a positive relationship between scoring high in sadism and being victimized by bullying (H_4_). This hypothesis appears since sadism has also been shown to be a personality predictor of victimization in other situations where the pleasure of inflicting pain can incur some costs (Pineda et al., 2020, 2021a). Finally, as being a perpetrator of bullying is related to suffering from it, we also anticipate that higher scores in the Dark Tetrad traits, mainly psychopathy and sadism, will predict higher victimization behaviors—in all the different victimization subtypes (*H*_5_).

## Materials and methods

### Participants and procedure

The sample consisted of 393 adolescents (53.7% male and 46.3% female) from four high schools in the Province of Alicante. The mean age of the participants was 14.18 years (*SD* = 1.30, range 12–18 years), and they were students in the first, second, third, and fourth years of compulsory secondary education and the first year of high school. Participants were able to complete the survey in two ways: on paper or in an online format through the DetectaWeb platform (Piqueras et al., 2017). The survey was carried out during the 2018–2019 and 2019–2020 academic years.

The project received approval from the university's ethics committee to carry out the study (Reference DPS.JPR.04.16). Participants were asked to submit an informed consent document signed by a parent.

### Measures

#### Peer bullying questionnaire (CAI)

The CAI is a Spanish self-report that measures bullying behavior among peers (Magaz et al., 2016). It includes two scales, the Bullying Behavior Scale (CAI-CA) and the Gender Bullying Behavior Scale (CAI-CAG), but in this paper, only the CAI-CA was used. It comprises 39 items, with seven subscales: physical abuse (e.g., they kick me), verbal abuse (e.g., they insult me), direct social exclusion (e.g., they stop me from playing with them), indirect social exclusion (e.g., they stop talking to me), threats (e.g., they threaten to tell you things about my family or me), cyberbullying (e.g., they send me cell phone messages or emails to insult or threaten me), and object-based aggression (e.g., they hit me with objects, for example, with sticks, scissors, rocks, etc.).

This instrument is answered on a Likert-type scale from 0 = *never* to 2 = *many times*. The reliability of scales in the original study ranged from 0.45 and 0.83 (Cronbach's Alpha: physical abuse = 0.79, verbal abuse = 0.83, direct social exclusion = 0.77, indirect social exclusion = 0.58, threats = 0.70, cyberbullying =0.45, and object-based aggression = 0.56 (Magaz et al., 2016).

#### Short Dark Triad

The Short Dark Triad (SD3) is a short self-reported instrument that measures the three personality traits of the Dark Triad: machiavellianism (e.g., I tend to manipulate people to get what I want), narcissism (e.g., people see me as a leader), and psychopathy (e.g., I tend to have no remorse; Jones and Paulhus, 2014). It consists of 27 items, with nine items per trait, that are answered on a Likert-type scale from 0 = *strongly disagree* to 4 = *strongly agree*. It has been validated in Spain, showing good psychometric properties (Cronbach's alpha: Machiavellianism = 0.73, narcissism = 0.61, and psychopathy = 0.68) (Pineda et al., 2020).

#### Assessment of sadistic personality

The Assessment of Sadistic Personality (ASP) is a 9-item unidimensional scale that measures everyday sadism (I have made fun of other people to let them know I am in control). It is answered on a Likert-type scale from 0 = *strongly disagree* to 4 = *strongly agree* (Plouffe et al., 2017). The original version shows adequate consistency, with a Cronbach's alpha of 0.83. The validation with a Spanish sample also shows adequate internal consistency indices, with a Cronbach's alpha and McDonald's Omega of 0.75 (Pineda et al., 2021b).

### Data analysis

Descriptive statistics were calculated using the SPSS statistical software (version 23; https://www.ibm.com/es-es/analytics/spss-statistics-software). Means and standard deviations were obtained to know the scores on each instrument administered to the participants. For the calculation of the internal consistencies (reliability of the instruments), SPSS and the statistical program Jamovi (version 1.6.23; https://www.jamovi.org/) were used, which offered the values of Cronbach's alpha and McDonald's Omega.

Correlations were also calculated with SPSS to know both the magnitude and the direction (positive or negative) of the relationships between the different variables. Regression models were also calculated with this statistical program to determine the predictive ability of the Dark Tetrad traits for the seven factors of bullying as victimization (criterion variables). Therefore, seven regression models were calculated; in the first block, the specific contribution of the sociodemographic variables (sex and age) was considered. The four Dark Tetrad traits were added in the second block to determine their influence. The percentages of the total variance explained (*SR*^2^) were calculated for each variable.

## Results

### Descriptive statistics and internal consistency of the instruments

Attending to the descriptive statistics (Table 1) of the Dark Tetrad traits, the sample of this study obtained the highest scores in narcissism and the lowest in sadism. In relation to the victimization variables, the sample obtained higher scores on the scales that measure victimization by physical and verbal aggression and lower scores on the scale that measures the possibility of being threatened.

**Table 1:** Means and standard deviations for response rates.

	**Total (*****N*** = **393)**				
	**Range of scores**	***M***	***SD***	**Cronbach's alpha**	**McDonald's omega**
**Dark Tetrad**					
Machiavellianism	0–36	11.33	5.47	0.73	0.75
Narcissism	0–36	14.20	4.78	0.55	0.59
Psychopathy	0–36	9.54	5.33	0.69	0.73
Sadism	0–36	5.72	5.81	0.81	0.87
**Bullying as victimization**					
Physical abuse	0–16	1.02	2.18	0.85	0.86
Verbal abuse	0–22	2.33	3.28	0.85	0.86
Direct social exclusion	0–10	0.46	1.25	0.78	0.80
Indirect social exclusion	0–8	0.59	1.11	0.53	0.73
Threats	0–8	0.09	0.45	0.60	0.63
Cyberbullying	0–8	0.12	0.59	0.73	0.76
Object-based aggression	0–6	0.16	0.57	0.59	0.60

All instrument factors showed acceptable and good internal consistency indices (with alphas and omegas ranging between 0.69 and 0.87), except for narcissism, threats, object-based aggression, and indirect social exclusion, with lower reliabilities (alphas and omegas between 0.53 and 0.63; see Table 1).

### Association between bullying as victimization and Dark Tetrad traits

The four Dark Tetrad traits presented significant positive correlations with bullying as victimization factors (see Table 2). Machiavellianism presented significant positive correlations with being physically and verbally abused and with being directly and indirectly excluded; narcissism, on the other hand, correlated positively with being physically abused (*p* < 0.05); and finally, sadism and psychopathy showed significant (*p* < 0.01) direct correlations with all the bullying as victimization dimensions.

**Table 2:** Bivariate correlations between dark tetrad personality traits and seven types of bullying victimization behaviors.

	**Machiavellianism**	**Narcissism**	**Psychopathy**	**Sadism**
Physical abuse	0.16^**^	0.11^*^	0.20^**^	0.23^**^
Verbal abuse	0.16^**^	0.06	0.24^**^	0.33^**^
Direct social exclusion	0.10^*^	−0.01	0.20^**^	0.26^**^
Indirect social exclusion	0.17^**^	0.01	0.21^**^	0.24^**^
Threats	0.04	0.02	0.13^**^	0.19^**^
Cyberbullying	0.05	0.05	0.13^**^	0.27^**^
Object-based aggression	0.04	0.04	0.13^**^	0.19^**^

* p < 0.05,

** p < 0.01.

### Predictive ability of the Dark Tetrad traits on bullying as victimization

Regarding the predictive ability of the Dark Tetrad traits for bullying as victimization, the interest lies in analyzing the influence of the traits for each of the seven bullying factors separately (see Table 3). The results showed that the sociodemographic variables (age and gender) in all seven models presented a null contribution (0%) of the total explained variance of bullying as a victimization factor. However, when Dark Tetrad traits were included in the models, the models became significant. These traits explained up to 14.00% (*p* = 0.001) of bullying victimization behaviors, with verbal abuse being the model with the highest contribution. Of the four dark traits, sadism stands out as the trait with the highest specific contribution (reaching *SR*^2^ = 7.67%).

**Table 3:** The predictive capacity of the Dark Tetrad for bullying as victimization.

**Criterion variable**	**Predictor variable**	**Step 1**				**Step 2**			
		**β**	***t***	***r_*x*.*y*_***	***sr* ^2^**	**β**	***t***	***r_*x*.*y*_***	***sr* ^2^**
Physical abuse	Age	−0.07	−1.23	−0.07	0.48%	−0.13	−2.31^*^	−0.13	1.56%
	Gender	0.10	1.8	0.10	1.04%	0.12	2.10^*^	0.11	1.28%
	Machiavellianism					0.07	0.94	0.05	0.26%
	Narcissism					−0.06	−0.94	−0.05	0.26%
	Psychopathy					0.14	1.88	0.01	1.04%
	Sadism					0.16	2.47^*^	0.13	1.77%
	*R^2^*	0.01				0.09			
	*F*	2.21				5.27^***^			
Verbal abuse	Age	0.02	0.37	0.02	0.04%	−0.05	−0.94	−0.05	0.24%
	Gender	0.10	1.74	0.10	0.94%	0.12	2.22^*^	0.12	1.37%
	Machiavellianism					−0.01	−0.11	−0.01	0.00%
	Narcissism					−0.10	−1.63	−0.09	0.74%
	Psychopathy					0.15	2.08^*^	0.11	1.21%
	Sadism					0.28	4.46^***^	0.24	5.52%
	*R^2^*	0.01				0.14			
	*F*	1.66				8.09^***^			
Direct social exclusion	Age	−0.04	−0.66	−0.04	0.14%	−0.09	−1.52	−0.08	0.69%
	Gender	0.01	0.21	0.01	0.01%	0.03	0.55	0.03	0.09%
	Machiavellianism					−0.07	−0.99	−0.05	0.29%
	Narcissism					−0.12	−1.9	−0.10	1.08%
	Psychopathy					0.18	2.43^*^	0.13	1.77%
	Sadism					0.18	2.73^**^	0.15	2.22%
	*R^2^*	0.01				0.07			
	*F*	0.23				3.95^**^			
Indirect social exclusion	Age	−0.01	−0.07	−0.01	0%	−0.05	−0.97	−0.05	0.28%
	Gender	0.09	1.52	0.09	0.72%	0.10	1.79	0.10	0.96%
	Machiavellianism					0.10	1.4	0.08	0.58%
	Narcissism					−0.14	−2.21^*^	−0.12	1.46%
	Psychopathy					0.14	1.86	0.10	1.04%
	Sadism					0.10	1.59	0.09	0.74%
	*R^2^*	0.01				0.07			
	*F*	1.52				3.85^**^			
Threats	Age	0.05	0.82	0.05	0.21%	0.01	0.15	0.01	0.01%
	Gender	0.04	0.67	0.04	0.14%	0.05	0.92	0.05	0.26%
	Machiavellianism					−0.12	−1.62	−0.09	0.81%
	Narcissism					−0.03	−0.50	−0.03	0.07%
	Psychopathy					0.13	1.66	0.09	0.83%
	Sadism					0.19	2.87^**^	0.16	2.5%
	*R^2^*	0.01				0.06			
	*F*	0.63				3.04^**^			
Cyberbullying	Age	0.09	1.52	0.09	0.72%	0.04	0.74	0.04	0.16%
	Gender	0.07	1.21	0.07	0.45%	0.08	1.51	0.08	0.66%
	Machiavellianism					−0.13	−1.86	−0.10	1.00%
	Narcissism					0.02	0.34	0.02	0.03%
	Psychopathy					0.01	0.09	0.01	0.00%
	Sadism					0.34	5.16^***^	0.28	7.67%
	*R^2^*	0.01				0.11			
	*F*	2.09				6.1^***^			
Object-based aggression	Age	0.12	1.87	0.10	1.08%	0.08	1.32	0.07	0.53%
	Gender	0.06	1.18	0.07	0.44%	0.08	1.36	0.08	0.56%
	Machiavellianism					−0.09	−1.23	−0.07	0.46%
	Narcissism					−0.02	−0.34	−0.02	0.04%
	Psychopathy					0.10	1.35	0.08	0.56%
	Sadism					0.14	2.16^*^	0.12	1.44%
	*R^2^*	0.02				0.05			
	*F*	2.7				2.61^*^			

* p < 0.05,

** p < 0.01,

*** p < 0.001.

More specifically, sadism is significantly (and positively) associated with six of the seven victimization variables (it predicts all variables except indirect social exclusion). In contrast, psychopathy is significantly associated (also positively) with only two of the victimization variables (it predicts verbal abuse and direct social exclusion). Narcissism is significantly associated with only one of the variables (it predicts indirect social exclusion), but, unlike the previous traits, it does so in a negative sense. Finally, machiavellianism is not significantly associated with any of the seven variables.

## Discussion

The main aim of this paper was to analyze the relationship between being victimized by bullying and the Dark Tetrad traits, delving into the specific contributions of machiavellianism, narcissism, psychopathy, and sadism in different ways of bullying victimization in Spanish adolescents.

Firstly, the results obtained regarding the relationships between the traits of the Dark Tetrad and the different factors of bullying as victimization report interesting findings. Even though the four traits are considered negative or antisocial, significant positive relationships have been found. This finding might be explained since some bullies are or can also be victims and vice versa. Some authors conclude that this may be due to the tendency of some victims to react to the aggressions suffered. Moreover, on many occasions, victims may not perceive themselves as aggressors when, in fact, they do engage in bullying behaviors (e.g., Lopes-Neto, 2005; Silva et al., 2012; Choi and Park, 2018; Reisen et al., 2019). A recent study concludes that another possible cause is the cognitive restructuring and moral disengagement that victims of bullying may undergo. Thus, these individuals might learn that aggression is an effective behavior, which would eventually cause them to also perform the bullying behaviors, but they might also misinterpret their own victimization, considering the aggressive behaviors as normal (Falla et al., 2022).

Regarding the links between the traits and the bullying dimensions, positive correlations were obtained between the seven factors that make up bullying victimization and psychopathy and sadism, supporting H_1_ and H_4_. Therefore, those who score high on psychopathy or sadism tend to also present higher scores in all the bullying victimization subtypes. In the case of machiavellianism, this positive correlation is found with fewer subtypes of bullying victimization. Therefore, H_2_ is partially supported. Finally, narcissism was correlated with one of the victimization subtypes (physical abuse), so H_3_ is rejected.

These results confront the findings of Gul and Fatima (2016), in which the only Dark Triad trait correlated with being a victim of bullying in a sample of girls was psychopathy. Similarly, they are also contrary to those found by Boele et al. (2017), who obtained no significant correlations with any of the Dark Triad traits. Moreover, the results are also inconsistent when comparing our results with those found in other studies with different sample populations (e.g., young adults or workers). Some studies have found correlations with the three traits of the Dark Triad (Parker, 2019; Hayes et al., 2021), while others only with Machiavellianism when analyzing the traits of the Dark Tetrad (Fernández-del-Río et al., 2021). In turn, other studies that have analyzed these relationships with some of the traits separately have also found positive correlations between presenting high scores in psychopathy or narcissism and being a victim of bullying (e.g., Fanti and Henrich, 2015; Backe and Dankvardt, 2018; Antoniadou et al., 2019; Despoti et al., 2021). In the case of the positive relationship between being a victim and having narcissistic traits, one study concluded that perhaps people with these traits become victims after having been bullies. They consider that they may have engaged in bullying behaviors to increase their social status, which would eventually have a counterproductive effect and, over time, place them in a victimized position (Fanti and Henrich, 2015). This could explain the positive relationship found in this study between narcissism and one of the factors of victimization (being a victim of physical abuse).

Secondly, regression models conducted to determine the predictive ability of the personality traits composing the Dark Tetrad for bullying as victimization factors also partially presented the expected results (*H*_5_). Overall, these personality traits explain up to 14.00% of bullying victimization behaviors, contrary to the findings presented by Gul and Fatima (2016). Although it is impossible to establish the causality of the relationship, it can be affirmed that some people who suffer certain bullying behaviors (especially verbal abuse and cyberbullying) tend to present higher scores in the Dark Tetrad personality traits. The Dark Tetrad trait that tends to predict the possibility of being victimized by bullying the most is everyday sadism. This finding replicates previous pointing to the everyday sadism trait as a personality factor that tends to be present in those who suffer from different antisocial behaviors (Pineda et al., 2021a,b). It might be explained due to the pleasure that people with high punctuations in sadism obtain from perpetrating these behaviors, which makes them not consider the consequences of being victimized in the same way (Foulkes, 2019).

Mixed results have been found regarding the predictive ability of the other Dark Tetrad traits. While psychopathy tends to be a positive predictor of all victimization behaviors, narcissism and Machiavellianism present different relationships. Previous studies have also shown psychopathy as a possible predictor of bullying victimization (Linton and Power, 2013; Parker, 2019). Perhaps, the callous personality of those with higher scores in psychopathy makes them less disturbed by suffering from bullying. Thus, they also continue their common behaviors as perpetrators in a dyadic relationship. On the other hand, although very weak, narcissism tends to predict not being victimized by almost all the bullying subtypes, which is congruent with the study by Fernández-del-Río et al. (2021). Narcissism, as in other contexts studied (e.g., with emotional intelligence, wellbeing, or civic engagement), is associated in the opposite direction to psychopathy and machiavellianism due to the way of behaving associated with their sense of entitlement or grandiosity (Rico-Bordera et al., unpublished^2^; Schreyer et al., 2021; Van Groningen et al., 2021). Finally, machiavellianism presents mixed findings with regard to the different victimization subtypes. Again, weak links were found. In summary, people with high scores in machiavellianism, convergent with their nature and with previous studies, tend to be slightly more involved in less observable behaviors such as indirect social exclusion and not be involved in more visible ones like threats or cyberbullying (Jones and Paulhus, 2014; Parker, 2019).

The results obtained in this study highlight the need for further research in this field, especially in adolescents. When an intervention is proposed, both bullies and victims are targeted (e.g., Ng et al., 2020; Martínez-Martínez et al., 2021). Thus, we propose that controlling or at least considering the personality traits that might be maintaining the victim's condition would be interesting.

To date, to our knowledge, only two studies have analyzed the traits of the Dark Triad (i.e., psychopathy, Machiavellianism, and narcissism) in adolescent victims of bullying (Gul and Fatima, 2016; Boele et al., 2017). In contrast to these studies, we have analyzed the association between Dark Tetrad traits (including every day sadism) and different victimization behaviors. Moreover, we analyzed it directly (i.e., without introducing other variables in the regression model). In the study by Gul and Fatima (2016), the association was controlled for the effect of bullying perpetration. In that study, bullying perpetration explains most of the variance because being a victim and perpetrating these behaviors tend to appear together (e.g., Choi and Park, 2018; Reisen et al., 2019) without considering the effect on the victimization of the personality variables alone.

### Limitations and future lines of research

This study presents several limitations. A first limitation implies, as previously stated, the low internal consistency of some of the factors (around 0.60). Specifically, the low internal consistency reliabilities are found in the dimensions measuring the narcissistic trait and those measuring being a victim of threats and object-based aggression. This could be due to the limitations that the scales used in this study could present (for example, problems in the wording or interpretation of the items). In addition, it is essential to note that self-reports, as is well known, have biases in their measurement, such as social desirability. Therefore, the participants may have slightly modified their answers due to social desirability (Abernethy, 2015; Althubaiti, 2016).

A second limitation relates to the low scores obtained in relation to having experienced bullying behaviors. Stronger relationships between the different constructs might be obtained by replicating this study with a larger sample of adolescents who have experienced bullying. Future research should replicate the present study with larger sample size and include more diverse samples to account for differences in negative personality traits by gender, race, socioeconomic status, and sexual orientation. As mentioned earlier in this study, certain groups of people may be more prone to bullying, such as people with low economic resources or people with a sexual orientation other than heterosexual (Fuentes Chacón et al., 2020; Kahle, 2020).

Likewise, it is likely that our findings cannot be generalized to other samples of different ages since. As mentioned throughout the discussion, our results differ from those obtained in other studies on young people or adults. Similarly, it may not be generalizable to other cultures since our results differ from those obtained in the two localized studies that analyzed these relationships with adolescents as we did (Gul and Fatima, 2016; Boele et al., 2017). In addition, Volk et al. (2018) already mentioned the importance of analyzing these relations in different cultures.

Finally, regarding these points, there is the last limitation of not having a longitudinal study, which would allow the establishment of the direction of causal relationships. As with the current design, it is not possible to state whether personality traits are a cause or consequence of bullying behaviors. For these reasons, it is considered necessary to replicate the study with a larger sample, which would guarantee the generalizability of the results. Furthermore, it would also be interesting to include a measure of bullying perpetration to help practitioners develop programs that consider the differences in personality of the agents involved in this behavior.

## Conclusion

Bullying remains a problem of great social relevance. For this reason, professionals in the field of psychology keep studying both the predictors of this problem and how to eradicate it. Knowing the personality traits of the people who carry out the bullying behaviors and those who suffer them is also an obvious need. Given this importance and the scarcity of studies that address it—especially those that analyze the personalities of bullied people—this is the first study that analyses the relationship between the darkest personality traits (i.e., the Dark Tetrad) and suffering from bullying in a Spanish sample.

A sociocultural shared belief is that “being bad” or presenting negative or dark personality tendencies usually determines malevolent behaviors, such as bullying, but it is less usual to expect that these same traits are related to suffering victimization. Given the cross-sectional nature of this study, it cannot be concluded with certainty whether victimization caused individuals to develop dark personalities or whether victims were already predisposed to these darker traits, but the need to know the personality of these victims is just as relevant as knowing the personality of bullies in order, for example, to design prevention and intervention programs aimed at all actors in bullying (perpetrators and victims). For now, this study shows that some of the traits of the Dark Tetrad, mainly sadism and psychopathy, are related to being a victim of bullying.

## Data availability statement

The datasets and analysis scripts used for this study can be found in the OSF repository: https://osf.io/ej74h/?view_only=8b0697f31f294479bd2e658ebee9bf97.

## Ethics statement

The studies involving human participants were reviewed and approved by Miguel Hernández University (reference DPS.JPR.04.16). Written informed consent was obtained from all participants' legal guardian/next of kin for their participation in this study.

## Author contributions

DP designed the study and oversaw all aspects of study implementation. JP acquired permissions for the research. AM-M and MG collected the data. MG managed the database. PR-B and JP performed the statistical analyses. PR-B wrote the first draft of the article. All authors reviewed the last version and approved the final manuscript.

## Funding

This study was funded by the Training of University Teaching Staff (FPU19/02233), a pre-doctoral contract funded by the Spanish Ministry of Universities.

## Conflict of interest

The authors declare that the research was conducted in the absence of any commercial or financial relationships that could be construed as a potential conflict of interest.

## Publisher's note

All claims expressed in this article are solely those of the authors and do not necessarily represent those of their affiliated organizations, or those of the publisher, the editors and the reviewers. Any product that may be evaluated in this article, or claim that may be made by its manufacturer, is not guaranteed or endorsed by the publisher.

## Footnotes

^1^Pineda, D., Martínez-Martínez, A., Galán, M.; Rico-Bordera, P., Piqueras, J. A., The Dark Tetrad and Online Sexual Victimization: The Guilty Sadism, unpublished.

^2^Rico-Bordera, P., Piqueras, J. A., Soto-Sanz, V., Rodríguez-Jiménez, T., Marzo, J. C., Galán, M., et al. (2021). Civic engagement and personality: the influence of the Big Five personality traits and the Dark Triad on engagement in civic behaviours. unpublished.
